# Dying at home in Belgium: a descriptive GP interview study

**DOI:** 10.1186/1471-2296-13-4

**Published:** 2012-01-19

**Authors:** Kathleen Leemans, Lieve Van den Block, Johan Bilsen, Joachim Cohen, Nicole Boffin, Luc Deliens

**Affiliations:** 1 Ghent University & Vrije Universiteit Brussel End-of-Life Care Research Group, Brussels, Belgium; 2 Department of Family Medicine, Vrije Universiteit Brussel, Brussels, Belgium; 3 Department of public health, Vrije Universiteit Brussel, Brussels, Belgium; 4 Scientific Institute of Public Health, OD Public Health and Surveillance, Brussels, Belgium; 5 EMGO Institute for Health and Care Research, VU University Medical Centre, Department of Public and Occupational Health, Amsterdam, The Netherlands

## Abstract

**Background:**

While increasing attention is being paid to enabling terminal patients to remain at home until death, limited information is available on the circumstances in which people at home actually die. Therefore this study aims to describe patient characteristics, functional and cognitive status and physical and psychological symptom burden in the last three months of life among Belgian patients dying at home, according to their GPs.

**Methods:**

In 2005, a nationwide and retrospective interview study with GPs took place on people dying at home in Belgium as reported by Sentinel Network of GPs in Belgium. GPs registered all deaths (patients aged 1 year or more) weekly and were interviewed about all patients dying non-suddenly at home, using face-to-face structured interviews.

**Results:**

Interviews were obtained on 205 patients (90% response rate). Between the second and third month before death, 55% were fully invalid or limited in self-care. In the last week of life, almost all were fully invalid. Fifty four percent were unconscious at some point during the last week; 46% were fully conscious. Most frequently reported symptoms were lack of energy, lack of appetite and feeling drowsy. Conditions most difficult for GPs to manage were shortness of breath, lack of energy and pain.

**Conclusions:**

Many people dying at home under the care of their GPs in Belgium function relatively well until the last week of life and cognitive status seems to be preserved until the end in many cases. However, symptoms which GPs find difficult to control still manifest in many patients in the final week of life.

## Background

With two thirds of all deaths in Belgium occurring non-suddenly or expectedly, mostly as a result of a serious chronic disease [1], safeguarding good quality of life at the end of life is important [2].

One way of improving the dying experience, as illustrated by the statements of the WHO [3], is to enable people to die at home under the care of their general practitioner (GP), as many would prefer [4,5]. While studies have shown that more than half of terminal patients prefer to die at home [6], in Belgium still only about a quarter of all deaths and 29% of cancer deaths occur there [7]. A recent study has shown that, despite the efforts being made, the percentage of people dying at home has not increased in the past decade [8].

While increasing attention is being paid to enabling patients to remain at home when receiving palliative care, limited information is available on how well and in what circumstances people actually die when they are able to stay at home [4]. Therefore it is important to have insight into the experience of dying at home, describing a patient's clinical, functional and cognitive status at the end of life and their symptoms and symptom burden [9-11].

GPs in Belgium, as in many countries, have built up a long-term relationship with their patients over the course of many years [12], making their role pivotal for patients spending most of their time at home at the end of life. Information on how well patients are dying at home and insights into what type of problems and type of patients GPs are confronted with are very important from a public health perspective as they can guide general practice and help caregivers to further develop and improve the care they deliver at the end of life.

This study has the following research questions:

- What are the socio-demographic and illness characteristics of patients dying non-suddenly at home in Belgium?

- How well do patients dying at home function physically and cognitively in the last three months and the last week of their lives?

- Which physical and psychological symptoms did patients dying at home find most burdensome in the last week of their lives according to the GP?

- What symptoms do GPs find most difficult to treat in the last week of life of patients dying at home?

## Methods

### Study design and participants

In 2005 and 2006 a large-scale mortality follow-back study was conducted to monitor end-of-life care and decision-making in Belgium using data from the SENTI-MELC study, the study on **M**onitoring **E**nd-of-**L**ife **C**are via the Nationwide **Senti**nel Network of General Practitioners in Belgium [13]. Since then, this registration study has been repeated every year. The network, representative of all Belgian GPs in terms of age, sex and region, proved to be a reliable surveillance system for health-related epidemiological data [14] and covers around 1.75% of the total Belgian patient population [15].

During the registration period a robust representative sample, not restricted to a specific setting, age group or disease, of non-sudden deaths of patients of one year or older (n = 1690) was identified by the GPs [16]. The 1690 deaths registered by the GPs were comparable in terms of age, sex and place of death to the deaths occurring within the general population [17].

**Figure 1:**
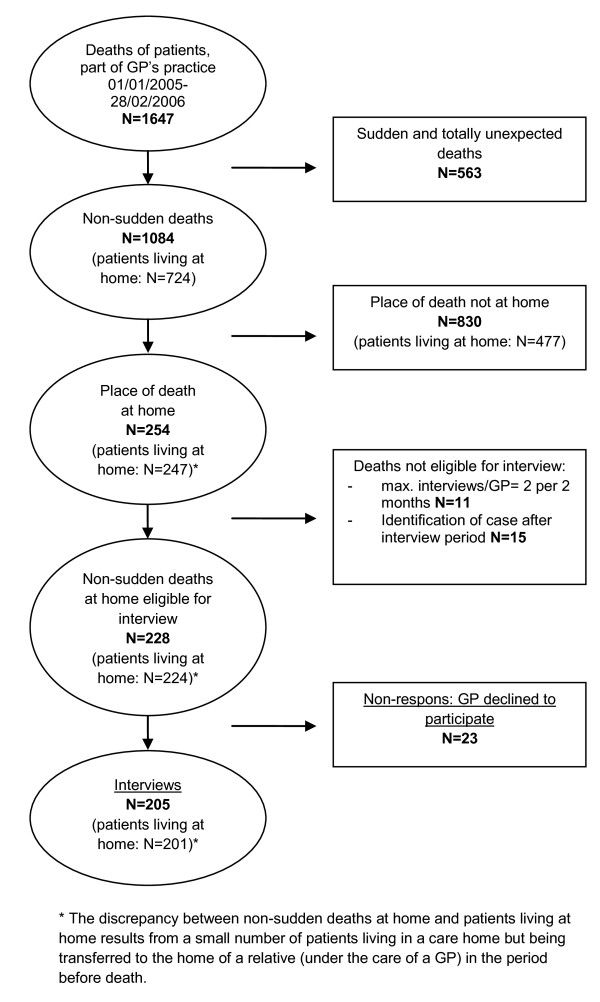
**Study population**.

To complement the data of the registration study with more detailed information about the end of life and end-of-life care of a relevant subsample of patients (ie those who died non-suddenly and expectedly in the fourteen months between January 2005 and February 2006 (n = 1647), an interview study was conducted with the GP of each one (see Figure 1).

A choice was made for a quantitative rather than a qualitative approach in the interview study and structured and standardized interviews were conducted in order to obtain both detailed information and standardized information, comparable across all patients. During the interviews we wanted to give the GPs the opportunity to explore some of the questions.

For this article which aims to describe the end-of-life circumstances of those dying at home, we selected only all interviews regarding those who died at home and whose death was, according to the GP, not 'sudden and totally unexpected' (n = 254). Of this sample, 247 were living at home before death and died there. The remaining 7 patients lived in a care home but were transferred to the home of a relative shortly before death, where they received end-of-life care from a GP (Figure 1). In this study dying at home is thus defined as dying at one's own home or dying at the home of a relative.

### Procedure

GPs registered weekly all deaths in their practice during the SENTI-MELC registration study, using a standardized form [13]. The GPs of patients meeting the interview inclusion criteria were contacted by telephone to request their participation in a face-to-face interview on the final phase of life. In order to prevent recall bias, the interview took place no longer than six months after the death of the patient. During the interview, GPs had access to electronic patient files. All interviewers were students of psychology or medicine and had followed training sessions in general interview techniques (training provided by one of the authors (LVDB)).

Patient names were never identifiable to the interviewers nor to other researchers involved: GPs used anonymous codes to refer to their patients in the registration form. The Ethical Review Board of Brussels University Hospital approved the study protocol and anonymity procedures (reference 2004045). Full description of the research protocol is found elsewhere [13].

### Measurements

The interview with the GP was face-to-face and structured with closed-ended questions and the use of optional 'other' categories. Patient characteristics, ie the socio-demographic information and care characteristics, were retrieved from standardized registration forms, completed by the GPs during the SENTI-MELC registration study and from the interview study. During the interview, GPs were asked about the patient's:

- illness characteristics ie diagnosis, comorbidities, duration of illness and cause of death

- functional status ie the Eastern Cooperative Oncology Group (ECOG) Performance Status during the second and third months before death, in the second to fourth week before death and in the last week of life [18]

- cognitive status in the last week of life ie the GP's judgment about the patient's level of consciousness, ability to communicate and ability to make decisions

- symptom burden in the last week of life ie Memorial Symptom Assessment Scale Global Distress Index (MSAS-GDI) which has been found to be a reliable and valid measure to assess global symptom distress. The GDI is the average of the frequency of four psychological symptoms (feeling sad, worrying, feeling irritable and feeling nervous) on a scale from 0 'never' to 4 'almost constantly' and the distress associated with six physical symptoms (lack of appetite, lack of energy, feeling drowsy, constipation, dry mouth and pain) on a scale from 0 'not at all' to 4 'very much' [19,20]. The tool has been adapted for retrospective administration by family respondents ('Family MSAS-GDI') [21] and for completion by professional healthcare providers, ie hospice care providers [20].

### Analysis

The data-entry into SPSS was done with consistency, range and skip checks and the registration forms were closely scrutinized for errors. All data were entered twice. Descriptive analyses were performed using SPSS 19.0 (SPSS Inc., Chicago, IL).

## Results

The GPs of 205 patients participated in the interview study, resulting in a participation rate of 90% (Figure 1). We compared our total study population of deaths at home (including the sudden deaths) with all deaths at home as reported through the death certificates for the whole of Belgium and found it representative in terms of age and gender. On the basis of death certificates we could, however, not select only the non-sudden and expected deaths occurring at home. Therefore we also compared the 129 deaths that occurred in Flanders or Brussels (out of the 205 non-sudden deaths occurring at home) with the non-sudden deaths occurring at home in a previous study in Flanders and Brussels, differentiating between sudden and non-sudden deaths with the same selection question. We found the sample in Flanders and Brussels to be representative of all deaths in the same regions (N = 595): no significant differences were found for age and gender (Binominal 95% CI, exact method) [22] (not shown in table). For the remaining 76 cases in our sample of deaths who died in the Walloon part of Belgium (40% of the population), no comparison data from other studies were available.

### Characteristics of patients and their illness

Of those dying under the care of their GPs 58% were between 65 and 84 years old. Approximately two thirds were male. Sixty five percent had a partner at time of death and 82% were living at home with one or more others. People dying at home were younger, more often male and more often living with a partner or with others (table 1) than those dying in a setting other than home. They were also more likely to have died from cancer.

**Table 1:** Characteristics of people dying non-suddenly at home* in Belgium

	Non-sudden deaths at home†		Non-sudden deaths not at home‡		
	**N = 205**	**%**	**N = 830**	**%**	**p-value§**

**Age (years)**					**.002**
1-64	35	18	83	10	
65-84	116	58	449	54	
85+	48	24	284	34	
**Gender, female**	77	38	456	55	**.000**
**Region**					.154
Flanders	114	56	520	63	
Wallonia	76	37	253	30	
Brussels	15	7	57	7	
**Educational level**					.323
Primary school or less	125	61	526	63	
Secondary school	40	20	144	17	
High school/university	19	9	55	7	
Unknown	21	10	105	13	
**Fixed partner at time of death**	132	65	341	41	**.000**
**Living at home before death**					**.000**
No||	4	2	350	42	
Yes, alone	33	16	148	18	
Yes, with one or more others	168	82	329	40	
**Financial status**					.401
(very) low	54	26	240	29	
Average	106	52	412	50	
(very) high	44	22	163	20	
**Cause of death**					**.000**
Cancer	118	58	291	35	
Non-cancer	81	40	534	64	

* Own home or home of a relative

† Missing values for non-sudden deaths at home: for Age n = 6, for Fixed partner at time of death n = 1, for Financial status n = 1, for Cause of death n = 6

‡ Missing values for non-sudden deaths not at home: for Age n = 14, for Fixed partner at time of death n = 5, for Living at home before death n = 3, for Financial status n = 15, for Cause of death, n = 5

§ p-value for Chi-square test

|| The 4 patients not living at home before death lived in a care home and were transferred to the home of a relative shortly before their death, where they received end-of-life care from a GP.

Sixty percent had cancer as the main diagnosis and 34% suffered additionally from heart complaints (table 2).

**Table 2:** Illness characteristics of deaths occurring non-suddenly at home*

Variable†	N = 205	%
**Main diagnosis**		
Cancer	122	60
Cerebral vascular accident	18	9
Heart failure	19	9
Old age	6	3
Dementia	9	4
Chronic obstructive pulmonary disease	11	6
Organ failure	7	3
Other	11	6
**(Occurrence of) Comorbidities **‡		
None	38	19
One or more	167	81
*Chronic obstructive pulmonary disease*	49	24
*Heart complaints*	70	34
*Diabetes*	20	10
*Hypertension*	51	25
*Joint arthritis*	38	18
*Other*	180	88
**Cause of death**		
Cancer	118	58
Cardiovascular disease	26	13
Pulmonary (respiratory) disease	16	8
Nervous system disease	14	7
Other/unknown	25	12
**When did illness start?**		
< 1 month before death	21	10
1-6 months before death	70	34
> 6 months before death	114	56
**When was patient diagnosed?**		
< 1 month before death	21	10
1-6 months before death	38	19
> 6 months before death	142	71

* Own home or home of a relative

† Missing answers: for Main diagnoses n = 2, for Cause of death n = 6, for Duration of illness until death n = 4, for Period until death after onset of diagnose n = 4

‡ Multiple answers possible

### Functional and cognitive status

Following the ECOG performance scale, 24% of people dying at home were fully invalid and 31% were limited in self-care between the second and the third month before death (table 3). In the last month this rose to 47% and 35% respectively. The percentage being fully active or limited in functioning dropped from 26% between the second and third month before death to 7% in the last month and to 1% in the last week of life.

**Table 3:** Functional and congnitive status of Belgian patients who die at home* non-suddenly

Variable†	N = 205	%
**Functional status between the 2^nd ^and 3^rd ^month before death†**		
Fully active (able to carry on all pre-disease performance without restriction)	16	8
Limited functioning (Restricted in physically strenuous activity but ambulatory and able to carry out work of a light or sedentary nature)	37	18
Ambulant (capable of all self care but unable to carry out any work activities. Up and about more than 50% of waking hours)	38	18
Limited in self care (Capable of only limited self care, confined to bed or chair more than 50% of waking hours)	64	31
Fully invalid (Completely disabled. Cannot carry on any self care. Totally confined to bed or chair)	49	24
**Functional status in the last month of life **(last week excluded) **‡**		
Fully active	4	2
Limited functioning	10	5
Ambulant	22	11
Limited in self care	72	35
Fully invalid	97	47
**Functional status in the last week of life†**		
Fully active	0	0
Limited functioning	2	1
Ambulant	5	2
Limited in self care	23	11
Fully invalid	175	86
**Level of consciousness during the last week of life**		
Never unconscious during the last week of life	95	46
Unconscious one or more hours before death	43	21
Unconscious one or more days before death	59	29
Unconscious during the whole last week before death	7	3
*If unconscious for one or more days before death*		
More than three days before death	14	22
On the three last days of life	16	25
On the two last days of life	14	22
On the last day of life	21	31
**Able to communicate during the last week of life§**		
Without limitations	81	40
With limitations	102	50
Not at all	19	10
**Able to make decisions during the last week of life§**		
Yes	115	57
Sometimes	23	11
No	65	32

* Own home or home of a relative

† Missing answers: for "Functional status 2 and 3 months before death" n = 1, for "Level of consciousness during the last week of life" n = 1, for "If unconscious during one or more days before death" n = 1, for "Able to communicate during the last week of life" n = 3, for "Able to make decisions during the last week of life" n = 2

‡ Functional status measured via ECOG performance scale

§When the patient was unconscious during one or more hours or days, the GP was asked to consider the ability for communication and decision making during the days the patient was fully conscious.

Twenty one percent were unconscious during one or more hours before death, 29% during one or more days. Of the 54% percent who were unconscious at some point during the last week, almost half (47%) were unconscious for three days or more.

Ninety percent of dying people were able to communicate without or with only a little limitation (albeit not necessarily up to the last day or during the whole of the last week) and 57% were able to make decisions in the last week of life according to the GP (albeit not necessarily up to the last day or during the whole of the last week).

### Most burdensome physical and psychological symptoms

People dying at home under the care of their GP had an average of six symptoms (sd = 2.49) as measured by the MSAS-GDI (table 4). The most frequently reported psychological and physical symptoms were lack of energy (91%), lack of appetite (86%), feeling drowsy (72%), pain (56%), shortness of breath (54%), feeling sad (51%) and worrying (46%). When asked how frequently the patients experienced each psychological problem, GPs scored feeling sad and worrying as frequently or almost constantly present for 69% of patients experiencing these symptoms. When asked how much distress each physical symptom caused the patient, GPs judged lack of energy as quite a bit to very much in 63% of cases, lack of appetite in 42% of cases, feeling drowsy in 27% of cases, having pain in 32% of cases and shortness of breath in 54% of cases. The mean Global Distress Index over all patients was 1.57 with a range of 0.07 to 3.76 (sd = 0.68).

**Table 4:** Psychological and physical symptoms in the last week of life of Belgian patients dying at home* non-suddenly: MSAS-GDI (N = 205)

**Psychological symptoms**	**Presence**	**How frequently did the patient experience the symptom? N (%)?‡**				**Physical or psychological symptom most difficult to manage**	**Most distressing physical or psychological symptom**
	**N (%)**^†^	Rarely	Occasionally	Frequently	Almost constantly	N (%) §||	N (%) §¶
Feeling sad	104 (51)	6 (6)	26 (25)	36 (35)	35 (34)	14 (9)	10 (6)
Worrying	95 (46)	4 (4)	25 (26)	35 (37)	30 (32)	5 (3)	8 (5)
Feeling irritable	62 (30)	7 (11)	25 (40)	20 (32)	9 (15)	4 (3)	2 (1)
Feeling nervous	88 (43)	8 (9)	38 (43)	28 (32)	14 (16)	5 (3)	4 (2)
**Physical symptoms**	**Presence**	**How much did the symptom distress/bother the patient? N (%)?‡**					
	**N (%)^†^**	**Not at all/A little**	**Somewhat**	**Quite a bit**	**Very much**		
**Lack of appetite**	177 (86)	75 (42)	26 (15)	43 (24)	31 (18)	19 (12)	8 (5)
**Lack of energy**	187 (91)	36 (19)	30 (16)	50 (27)	67 (36)	30 (19)	47 (27)
**Pain**	114 (56)	37 (32)	38 (33)	24 (21)	13 (11)	18 (12)	28 (16)
**Feeling drowsy**	147 (72)	66 (45)	36 (24)	33 (22)	7 (5)	6 (4)	4 (2)
**Constipation**	78 (38)	34 (44)	28 (36)	11 (14)	4 (5)	6 (4)	6 (3)
**Dry mouth**	97 (47)	31 (32)	29 (30)	28 (29)	7 (7)	6 (4)	10 (6)
**Shortness of breath**	111 (54)	25 (23)	25 (23)	25 (23)	35 (31)	42 (27)	46 (27)
**Mean N° of symptoms per patient (SD)**	6.15 ± 2.49						
Global Distress Index (Mean, SD)**	1.57 ± 0.68						

* Patients dying at their own home or at the home of a relative; all percentages are column percentages except these about frequency of experience and distress of the symptoms which are row percentages (% of those patients whose GP answered yes to a symptom)

† missing values: for Feeling sad n = 24, for Worrying n = 24, for Feeling irritable n = 17, for Feeling nervous n = 16, for Lack of appetite n = 14, for Lack of energy n = 13, for Pain n = 12, for Feeling drowsy n = 14, for Constipation n = 34, for Dry mouth n = 28, for Shortness of breath, n = 11

‡ missing values: for Feeling sad n = 1, for Worrying n = 1, for Feeling irritable n = 1, for Lack of appetite n = 2, for Lack of energy n = 4, for Pain n = 2, for Feeling drowsy n = 5, for Constipation n = 1, for Dry mouth n = 2, for Shortness of breath n = 1

§ missing values: for Symptom most difficult to manage n = 32, for Most bothersome symptom n = 13

|| category 'other' n = 14, category 'none' n = 4

¶ category 'other' n = 14, category 'none' n = 5

** 18 cases excluded from analyses: for 18 cases the GP could not score > 3 symptoms

### Symptoms most difficult to treat

Most difficult of all physical and psychological symptoms to manage for GPs were shortness of breath (27%), lack of energy (19%) and pain (12%). The latter were seen as the most distressing for the patient in respectively 27%, 27% and 16% of cases.

## Discussion

This nationwide study of dying at home under the care of the GP in Belgium shows that 55% of patients dying at home were fully invalid or limited in self care at some point in the 2^nd ^and the 3^rd ^months before death, while the remaining were still minimally ambulant. In the last week of life, almost every patient was fully invalid or limited. Furthermore 54% were unconscious at some point during the last week. However, 46% were fully conscious during that whole last week, 90% were able to communicate without or with only a little limitation and 57% were able to make decisions, according to the GP. Most patients experienced lack of energy, lack of appetite and feeling drowsy in the last week of life. The most frequently occurring psychological symptoms, according to the GP, were feeling sad and worrying; physical symptoms scored by the GP as most distressing for the patients were lack of energy, shortness of breath and lack of appetite. Shortness of breath, lack of energy and pain were most difficult to manage for GPs.

This is, as far as the authors know, the first study describing the characteristics and circumstances surrounding the death of people dying at home in Belgium. This study used a nationwide representative surveillance network of GPs to identify a representative subset of non-sudden deaths at home in Belgium. The sample was not restricted to a specific age group or disease [23]. Further, it was based on a strong study design, using a large-scale retrospective registration study to identify a subgroup of patients for the interview study [13]. We obtained a high participation rate and the standardized and extensive face-to-face interviews with GPs were conducted within six months of death by trained interviewers.

The retrospective approach of the interview study implies a possible memory bias on the part of the GPs; their judgment about the patient's symptoms might also be biased to some extent, and thus results reflect the GP's interpretation. The study is also restricted to quantitative data; in depth qualitative exploration of patient or care characteristics was not possible. Finally, place of death of patients included in this study was limited to home, which makes it not representative for the whole of primary care in Belgium which also includes those being transferred to an institution at the very end of life as well as those residing in a care home [24].

This study shows that many patients dying at home maintained relatively good functional status in the last three months of life until the last week before death, when functional status significantly deteriorated. The majority of people dying at home were able to communicate without or with only a little limitation and make decisions in the last week of life, according to their GPs. This does not mean that they were able to do so at all times during the last week (eg only during moments of consciousness) or until the last day before death. While more than half of patients were unconscious at some point during the last week of life most of them were unconscious for two days or less. These results might also be associated with the disease trajectory, since six out of ten suffered from cancer, an illness characterized by a relatively short period of evident decline at the end of life. Other studies have also shown that functional decline is associated with the setting in which people died. Teno et al. (2001) [10] showed that people dying of cancer in the US and having a less impaired functional status were more likely to die at home.

The study also shows that patients do experience a number of distressing symptoms at the end of life when dying at home, according to their GPs. Many of the symptoms identified here were also found in other studies with terminal patients in other settings [25-27] ie lack of energy, lack of appetite, feeling drowsy, pain and shortness of breath. Moreover, shortness of breath and lack of energy can be associated with fatigue, ie an important but difficult to treat problem in palliative care [28], as is also shown in other studies focusing on the relationship of this symptom to time to death. Cheung et al. (2009) [29] in particular show the close relationship between time to death and the cluster of symptoms lack of appetite, drowsiness and fatigue among cancer patients. Although these patients are likely to have a lower symptom burden than those dying in the hospital or in a palliative care unit, the symptom burden of people dying at home in this study is still high. Home caregivers can use this information to improve their practice at the end of life by giving further attention to the treatment of these specific symptoms near death.

Interestingly, the symptoms that GPs most frequently indicated to be most difficult to manage were also those they most frequently indicated as most distressing of all physical and psychological symptoms ie shortness of breath, lack of energy and pain. Important here is that pain was mentioned as one of the most distressing of all physical and psychological symptoms for patients according to the GP, indicating GPs still consider pain as one of the most important symptoms experienced by people nearing death [30]. Additionally, 56% of the patients experienced pain in some way according to the GP, suggesting room for improvement of the treatment of pain in primary care alongside other frequently-occurring symptoms.

## Conclusions

To conclude, the results of this study emphasize that most people dying at home under the care of their GPs in Belgium function relatively well in the last months of life. In the last week of life, cognitive status is mainly preserved while functional status significantly deteriorates and many patients experience a relatively high symptom burden. The treatment of pain at the end of life remains one important concern for GPs while other physical and psychological symptoms such as feeling sad, shortness of breath or lack of energy also tend to be important issues in general practice. This makes thorough assessment, improved support and specialized training for the treatment of these specific symptoms important in improving the quality of dying at home.

## Competing interests

The authors declare that they have no competing interests.

## Authors' contributions

KL performed the statistical analysis and drafted the manuscript. LVDB conceived of the study, participated in its design and coordination and helped to draft the manuscript. JB participated in the design of the study and revised the article critically for important intellectual content. JC revised the article critically for important intellectual content. NB revised the article critically for important intellectual content. LD participated in the design of the study and revised the article for major intellectual content. All authors read and approved the final manuscript.

## Pre-publication history

The pre-publication history for this paper can be accessed here:

http://www.biomedcentral.com/1471-2296/13/4/prepub
